# Tuning of AKT-pathway by Nef and its blockade by protease inhibitors results in limited recovery in latently HIV infected T-cell line

**DOI:** 10.1038/srep24090

**Published:** 2016-04-14

**Authors:** Amit Kumar, Wasim Abbas, Laurence Colin, Kashif Aziz Khan, Sophie Bouchat, Audrey Varin, Anis Larbi, Jean-Stéphane Gatot, Kabamba Kabeya, Caroline Vanhulle, Nadège Delacourt, Sébastien Pasquereau, Laurie Coquard, Alexandra Borch, Renate König, Nathan Clumeck, Stephane De Wit, Olivier Rohr, Christine Rouzioux, Tamas Fulop, Carine Van Lint, Georges Herbein

**Affiliations:** 1 Department of Virology, Pathogens & Inflammation Laboratory, University of Franche-Comté and COMUE Bourgogne Franche-Comté University, UPRES EA4266, SFR FED 4234, CHRU Besançon, Besançon, France; 2 Laboratory of Molecular Virology, Institut de Biologie et de Médecine Moléculaires (IBMM), Université Libre de Bruxelles (ULB), Gosselies, Belgium; 3 Department of Medicine, University of Sherbrooke, Sherbrooke, Canada; 4 Department of Infectious Diseases, CHU St-Pierre, ULB, Bruxelles, Belgium; 5 Research Group “Host-Pathogen Interactions”, Paul-Ehrlich-Institute, Langen, Germany; 6 Immunity and Pathogenesis Program, Sanford Burnham Prebys Medical Discovery Research Institute, La Jolla, CA; German Center for Infection Research (DZIF), Langen, Germany; 7 Institut de Parasitologie et Pathologie Tropicale, University of Strasbourg, Strasbourg, France; 8 Department of Virology, Paris University, EA7327 Paris Descartes, APHP Necker Hospital, Paris, France

## Abstract

Akt signaling plays a central role in many biological processes, which are key players in human immunodeficiency virus 1 (HIV-1) pathogenesis. We found that Akt interacts with HIV-1 Nef protein. In primary T cells treated with exogenous Nef or acutely infected with Nef-expressing HIV-1 *in vitro*, Akt became phosphorylated on serine^473^ and threonine^308^. *In vitro,* Akt activation mediated by Nef in T-cells was blocked by HIV protease inhibitors (PI), but not by reverse transcriptase inhibitors (RTI). *Ex vivo,* we found that the Akt pathway is hyperactivated in peripheral blood lymphocytes (PBLs) from cART naïve HIV-1-infected patients. PBLs isolated from PI-treated patients, but not from RTI-treated patients, exhibited decreased Akt activation, T-cell proliferation and IL-2 production. We found that PI but not RTI can block HIV-1 reactivation in latently infected J-Lat lymphoid cells stimulated with various stimuli. Using luciferase measurement, we further confirmed that Nef-mediated reactivation of HIV-1 from latency in 1G5 cells was blocked by PI parallel to decreased Akt activation. Our results indicate that PI-mediated blockade of Akt activation could impact the HIV-1 reservoir and support the need to further assess the therapeutic use of HIV-1 PI in order to curtail latently infected cells in HIV-1-infected patients.

The serine/threonine kinase Akt (or protein kinase B) is a key regulator in the phosphoinositide 3-kinase (PI3K) signaling pathway and plays important roles in many cellular processes such as cell survival, metabolism, growth, and proliferation that are involved in HIV-1 pathogenesis^1^,^2^. Immune hyperactivation, a hallmark of HIV-1 infection, fuels the progression of the disease and could be critical for the formation of HIV-1 reservoirs in infected individuals^3^,^4^.

HIV-1 Nef is a multifunctional viral accessory protein without enzymatic activity that is abundantly expressed early in infection and has been shown to play an important role in numerous aspects of viral pathogenesis^2^,^5^,^6^. Viral pathogenesis and replication is largely attenuated in individuals infected with Nef deficient HIV-1 7. Endogenous Nef governs the downregulation of CD4+ receptor, major histocompatibility class (MHC) I, MHC II, CD80 and CD86 molecules in infected cells and interferes with several signaling pathways^8^.

Infection of CD4+ T cells by HIV-1 is largely influenced by their activation state. In activated CD4+ T cells, HIV-1 can readily undergo robust replication whereas resting CD4+ T cells are usually refractory to infection^9^. A number of studies suggest that Nef can reduce the threshold for CD4+ T cell activation, and Nef has been shown to interact with T-cell receptor and various downstream effectors or secondary messengers including Lck, LAT, lipid rafts, PI3K and Ca^2^,^8^,^10^,^11^.

Besides endogenously produced Nef, extracellular Nef protein is found in the serum and cerebrospinal fluid of infected individuals and displays immunomodulatory effects such as the suppression of immunoglobulin class switching in bystander B cells^12^,^13^,^14^. PI3K/Akt pathway, a key pathway of cell survival is known to be manipulated by a plethora of viral pathogens including human cytomegalovirus, Epstein Barr virus, influenza A virus and HIV-1 15. Few studies also reported the impact of protease inhibitors on Akt signaling in several cell types and in a clinical trial^16^,^17^,^18^,^19^. We hypothesized that the Akt pathway could play a role in HIV-1 reactivation. We found that the Nef protein participates to the hyperactivation of T cells through Akt activation and that blocking Akt activation could limit HIV-1 recovery from latently infected T cells.

## Results

### Exogenous Nef enters into PBLs and increases Akt phosphorylation

**Figure 1:**
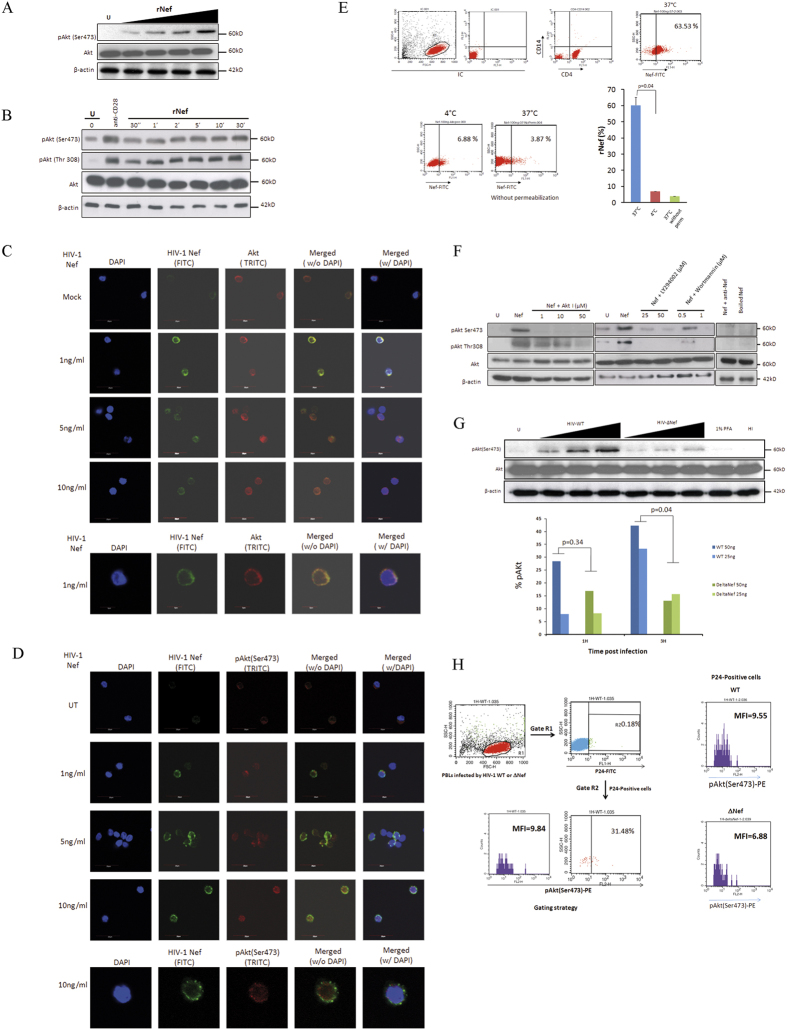
HIV-1 Nef is internalized by CD4 + T cells and activates Akt in PBLs which is mediated via PI3K in a dose and time dependent manners. (**A**), Dose-dependent (n = 3) and (**B**), Time-dependent activation of Akt (pAkt(Ser473)) in PBLs treated with rNef (n = 3). (**B**) Five million PBLs were either left untreated or treated with rNef (100 ng/ml) for various period of time (30 seconds to 30 minutes). As a positive control, PBLs were treated with anti-CD28 antibody. Expression of pAkt (Ser473, Thr308), total Akt and β-actin were detected by standard western blotting method as described in materials and methods (n = 3). (**C**,**D**) A series of confocal images showing internalization and colocalization of HIV-1 Nef and Akt (**C**) at serum concentration of Nef (1 to 10 ng/ml) and a dose response of rNef treatment on Akt activation (**D**) in PBLs isolated from healthy donors. (**E**), Internalization of rNef by CD4+ T cells determined by flow cytometry. Five million CD4+ T cells were treated with rNef for 30 min at 37 °C and 4 °C with and without permeabilization. Expression of rNef was determined by confocal microscopy (n = 3).(**F**), Activation of Akt in PBLs treated with rNef is mediated via PI3K. Western blot detection of activated pAkt (Ser473, Thr308) in the lysates derived from 5 × 10^6^ PBLs treated with 100 ng/ml of Nef with or without Akt (Akt inhibitor VIII) and PI3K inhibitors (LY294002 and Wortmannin) (n = 3). (**G**), Akt activation in PBLs by wild-type HIV-1, but not by isogenic HIV-1∆Nef. Five million PBLs were infected with various doses (of p24) of either wild type HIV-1 or ∆Nef virus infectious clones. Post infection lysates were prepared and expression of pAkt(Ser473), was determined by western blotting (upper panel) and flow cytometry (n = 2) (lower panel). (**H**), Left panel: Gating strategy for flow cytometry analysis. In this sample gating cells were first gated for PBLs (Gate R1). R1 gate is further analysed for p24 positive PBLs (Gate R2). Gate R2 is analyzed for the expression of pAkt. Right panel is the representative data of pAKt positive cells in p24 positive population obtained by infection of PBLs with WT and Delta Nef virus.

We studied the impact of Nef on Akt activity, which is activated by its phosphorylation on Ser473 and Thr308 residues^20^,^21^. We observed that treatment of PBLs with rNef led to Akt phosphorylation (pAkt) in a dose dependent (Fig. 1A) and time dependent manner as determined (Fig. 1B) by western blotting and confocal microscopy (Fig. 1C,D). We did not find any significant toxicity of rNef (1–100 ng/ml) for as long as 30 min as determined by Wst-1 cell viability assay (Supplementary Fig. 1A,B). We observed rapid phosphorylation of Akt by exogenous Nef suggesting the involvement of receptor mediated signaling^22^,^23^.

We further investigated the internalization of rNef by PBLs and its cellular colocalization with Akt. We assessed the purity of PBLs using CD14/45, CD8+ and CD4+T staining by flow cytometry (Supplementary Fig. 2). We incubated PBLs isolated from healthy donors with varying concentration of Nef (1–10 ng/ml) and determined the expression of Nef and Akt by immunofluorescence using confocal microscopy. We detected the expression of Nef both at the cell margins and in the cytoplasm (Fig. 1C). Expression of Akt was found both in cytoplasm as well as in the nucleus (Fig. 1C). In addition, we also observed the colocalization of internalized rNef and Akt mostly at the cell margins. Taken together our results indicate Nef is internalized by PBLs and colocalized with Akt (Fig. 1C).

To further demonstrate the internalization of rNef by PBLs, we incubated CD4+ T cells with rNef for 30 min at 37 °C and 4 °C under permeabilized and non permeabilized conditions. We found that rNef is internalized by CD4+ T at 37 °C and very little internalization was observed at 4 °C (Fig. 1E). To our knowledge this is the first report where internalization of rNef by PBLs has been demonstrated.

Phosphorylation of Akt on Ser473 and Thr308 was blocked by Akt inhibitor VIII (AktI) in PBLs treated with rNef (Fig. 1F). Since Akt activation is partly mediated by PI3K products such as PIP3, we determined whether the effect of HIV-1 Nef on Akt phosphorylation was dependent on cellular PI3K activity. The two PI3K inhibitors LY294002 and Wortmannin hindered the phosphorylation of Akt at Ser473 and Thr308 observed after HIV-1 rNef treatment, demonstrating that endogenous PI3K activity is required for Nef-mediated Akt activation (Fig. 1F). We did not detect any significant cytotoxicity of Akt and PI3K inhibitors used after two hours of treatment (data not shown).

Akt pathway plays a key role in cell survival and homeostasis^1^. Therefore, there are only limited numbers of phosphoproteins unaltered by Akt inhibitors. Phosphorylation of proteins upstream of Akt pathway is generally unaltered upon Akt inhibitors treatment such as focal adhesion kinase (FAK) and proline-rich tyrosine kinase 2 (PYK2). Therefore, to determine the specificity of the AktI inhibitor, we have determined for the phosphorylation level of FAK using western blot. We did not observe any significant change in the levels of pFAK in response to AktI treatment post 2 hours of treatment (Supplementary Fig. 3).

Nef is known to play important role in promoting viral infectivity in a host cell dependent manner in several independent studies^24^,^25^,^26^,^27^. Importantly Nef is associated with virion particles and therefore merely entry of the viral particles and hence Nef could activate Akt. We infected PBLs with wild type HIV-1 (HIV-WT) and an isogenic mutant infectious HIV-1 clone deleted for the *nef* gene (HIVΔNef) and compared the level of Akt activation. Wild-type HIV-1 infection was associated with an increased Akt phosphorylation in contrast to PBLs infected with HIVΔNef and the effect was dose dependent (Fig. 1G, upper panel). Since the effect of Nef on Akt activation would be more accurately analyzed in the fraction of infected cells, we infected PBLs with different amount of viral particles derived from wild type and Nef deleted mutant (based on their p24 quantitation) and measured the levels of pAkt at early time points only in the p24-positive cells (Fig. 1G lower panel, Fig. 1H). The gating strategy is shown in Fig. 1H, left panel. We found that with equal amount of virus particle, levels of pAkt were higher in WT at post 3 h of infection as compared to Nef-deleted mutant in p24-positive cells (p = 0.04 WT vs. Nef-deleted mutant) (Fig. 1G lower panel). In addition, we also observed relatively higher amount of p24 detection in PBLs infected with wild type HIV-1 as compared to delta Nef mutant post 24h of infection in unstimulated condition further confirms the decreased infectivity of delta Nef HIV-1 (Supplementary Fig. 4)

### HIV-1 Nef interacts with Akt and PI3K via its C and N terminal extremity respectively

**Figure 2:**
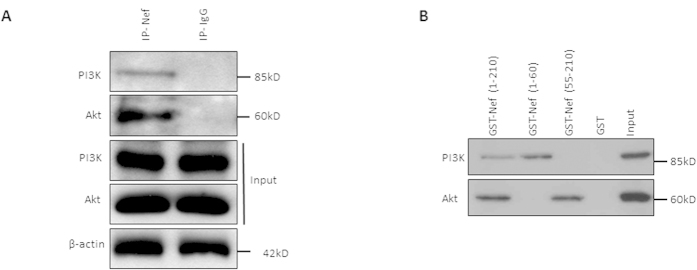
HIV-1 Nef interacts with Akt and PI3K. (**A**), Nef interacts with Akt and PI3K in PBLs treated with rNef as detected by co-immunoprecipitation. Five million PBLs isolated from healthy donor were left untreated or treated with HIV-1 rNef (100 ng/ml) and subjected to immunoprecipitation with anti-HIV Nef-Ab or isotype control antibody followed by western blot using indicated antibodies (n = 3). (**B**), Nef interacts with Akt and PI3K via its C-terminus and N-terminus respectively, as measured by pull-down assays. Representative western blot analysis of pull down assay using 1500 μg of PBLs lysates with 20 μg of different HIV-Nef constructs [N-terminus, 1-60aa, C-terminus 55-210aa and full length Nef, 1-210 aa] expressed in BL21DE as indicated. Input corresponds to 10% of the material used for pull down. Presence of PI3K and Akt was detected by respective antibodies mentioned in Material and Method section (n = 3).

We tested the interaction between Nef and Akt as well as between Nef and PI3K, an upstream kinase involved in Akt phosphorylation and known to interact with Nef^10^,^20^. We observed that endogenous Akt and endogenous PI3K co-immunoprecipitated with HIV-1 Nef in peripheral blood lymphocytes (PBLs) isolated from healthy donors treated with recombinant Nef (rNef) (Fig. 2A). To determine the domain of HIV-1 Nef that binds Akt or PI3K, we expressed the full-length HIV-1 Nef and Nef proteins truncated at their N-terminal or C-terminal extremity as GST fusion proteins in *E. coli* and tested their ability to interact with Akt and PI3K present in PBL lysates (Fig. 2B). Our GST pull-down assays indicated that HIV-1 Nef interacts with Akt via its C-terminal region (residues 55–210), while HIV-1 Nef interaction with PI3K occurs via Nef N-terminal region (residues 1–60) (Fig. 2B).

### Nef triggers the activation of NF-ĸB and CD28RE via lipid raft mediated Akt signaling

**Figure 3:**
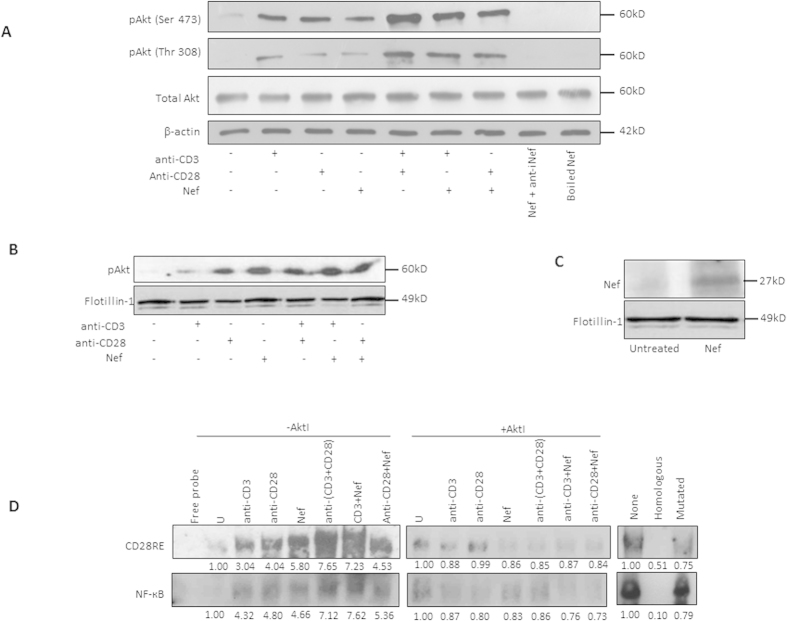
Nef triggers the activation of NF-ĸB and CD28RE via lipid raft mediated Akt signaling. (**A**), Exogenous Nef activates Akt in TCR stimulated PBLs as measured by the detection of pAkt(Ser473). Five million PBLs were either left untreated or stimulated in various combinations: rNef only, anti-CD3-mAb only, anti-CD3-mAb + rNef (100 ng/ml), anti-CD28-mAb only, anti-CD28-mAb + rNef (100 ng/ml) and anti-CD3-mAb + anti-CD28-mAb) for 30 min. Expression of pAkt(Ser473 Thr308), total Akt and β-actin was determined by western blotting (n = 2). As control boiled rNef and rNef incubated with anti-Nef antibody were also included. (**B**,**C**), Detection of pAkt(Ser473) (**B**) and rNef (**C**) in lipid raft fractions isolated from PBLs treated with rNef. Five million PBLs were either left untreated or stimulated in various combinations: rNef only, anti-CD3-mAb only, anti-CD3-mAb + rNef (100 ng/ml), anti-CD28-mAb only, anti-CD28-mAb + rNef (100 ng/ml) and anti-CD3-mAb + anti-CD28-mAb) for 30 min. Cell lysates were prepared in 1% triton X-100 and subjected to ultracentrifugation over a sucrose gradient. Lipid rafts fractions were isolated and expression of pAkt (Ser 473), Nef and flotillin-1 were determined by western blotting (n = 5). Flotillin-1 was used as a lipid raft associated marker. (**D**), Akt-dependent NF-κB and CD28RE activation in PBLs treated with rNef. Five million PBLs were either left untreated or treated with rNef (100 ng/ml) for 30 min in the presence and absence of TCR stimulation (anti-CD3-mAb, anti-CD28-mAb), and pre-treated or not with the Akt inhibitor VIII for 2 hrs. Electrophoretic mobility shift assays was performed to measured CD28 responsive element and NF-kB activation in response to stimuli on a 6% native polyacrylamide gel as described in the materials and methods (n = 3).

PI3K activity and consequently Akt activation contribute to T-cell receptor (TCR) signaling as key regulators of T-cell activation, differentiation and proliferation^28^,^29^,^30^. Therefore, we tested the effect of rNef on Akt phosphorylation in the context of TCR stimulation using treatment with anti-CD3 and/or anti-CD28 monoclonal antibodies. Recombinant Nef-mediated Akt phosphorylation was increased following TCR stimulation, compared to that observed upon rNef treatment alone (Fig. 3A). Our results indicate that rNef, alone or in conjunction with TCR stimulation is able to significantly activate Akt in PBLs from healthy donors. Since both proteins have been reported to translocate to lipid rafts^1^,^31^,^32^, we questioned whether the HIV-1 Nef/Akt interaction involves lipid rafts. We observed that rNef treatment alone or combined with TCR stimulation indeed induced the translocation of activated Akt to lipid rafts in parallel to its own localization into lipid rafts (Fig. 3B,C). In addition, we observed the colocalization of rNef and pAkt at the cell margins further suggesting lipid rafts as a possible site of Nef and Akt interaction (Fig. 1C,D).

**Figure 4:**
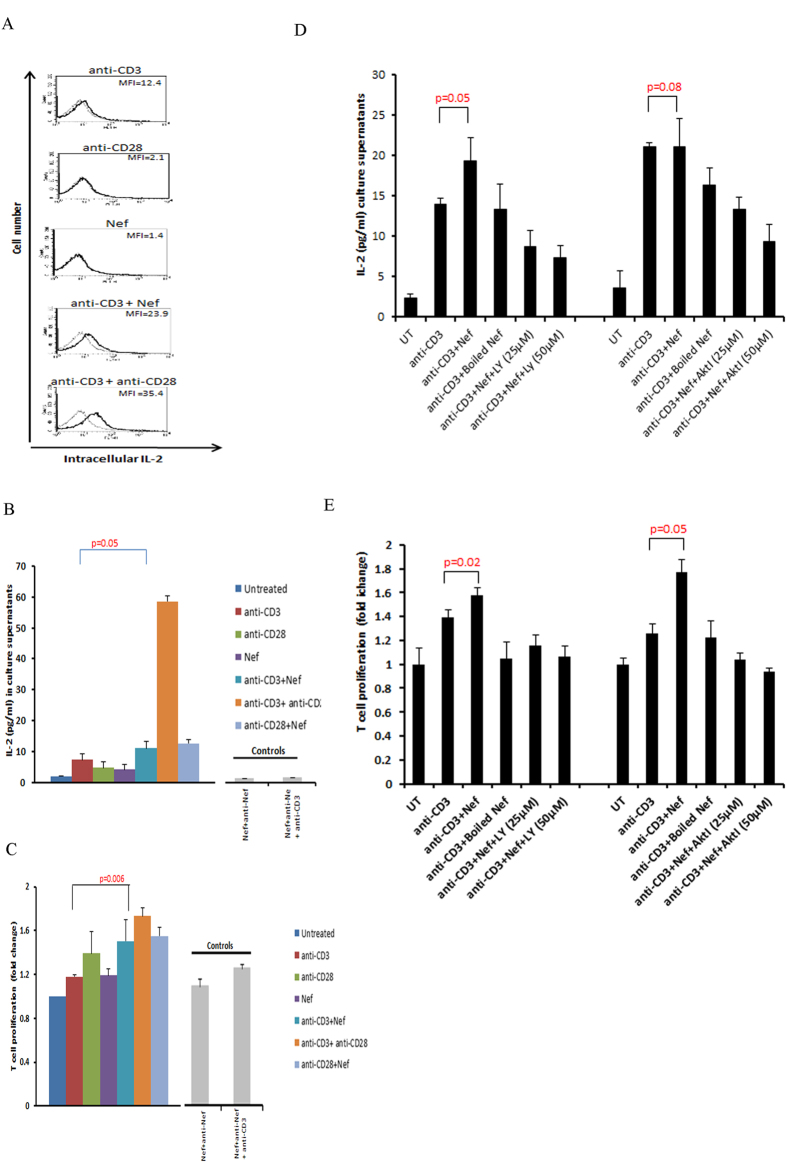
*In vitro* rNef-mediated Akt-dependent hyperactivation of T cells favors increased IL-2 production and T-cell proliferation. (**A**,**B**) Exogenous HIV-1 Nef triggers IL-2 production in TCR-stimulated T-cells. (**A**), Five million PBLs were stimulated with anti-CD3-mAb (5 μg/ml), anti-CD3-mAb + rNef, anti-CD28-mAb (5 μg/ml), anti-CD28-mAb + anti-CD3-mAb or rNef (100 ng/ml) alone for 24 h. Post treatment cells were fixed, permeabilized and expression of intracellular IL-2 was determined by flow cytometry. Flow cytometric data indicating the mean fluorescence intensity (MFI) for intracellular IL-2 production. Data are representative of 5 independent experiments. (**B**), Five million PBLs were either left untreated or treated with rNef (100 ng/ml), anti-CD3-mAb (5 μg/ml), anti-CD28-mAb (5 μg/ml), anti-CD3-mAb + rNef, anti-CD28-mAb + rNef, and antiCD3-mAb + antiCD28-mAb for 24 h. Post 24 h supernatants were collected and IL-2 production was measured in supernatants by ELISA. Means ± s.d. (n = 3). (**C**), Exogenous HIV-1 Nef triggers cell proliferation in TCR-stimulated T cells. Five million PBLs were either left untreated or treated with rNef (100 ng/ml), anti-CD3-mAb (5 μg/ml), anti-CD28-mAb (5μg/ml), anti-CD3-mAb + rNef, anti-CD28-mAb + rNef and antiCD3-mAb+ antiCD28-mAb in the absence of Akt I and PI3K inhibitor for 24 h. Cell proliferation was measured using MTT cell proliferation assay. Means ± s.d. (n = 3). (**D**,**E**), Exogenous HIV-1 Nef triggers IL-2 production and T cell proliferation in CD3 stimulated cells in PI3K/Akt dependent manner. Five million PBLs were either left untreated or treated with Akt I (25 μM, 50 μM) and PI3K inhibitor LY294002 (25 μM, 50 μM) for 2 h followed by treatment with rNef (100 ng/ml), heat denatured rNef (100 ng/ml), anti-CD3-mAb (5 μg/ml) and anti-CD3-mAb + rNef, for 24 h. After 24 h supernatants were collected and IL-2 production and T cell proliferation was determined by ELISA and MTT assay respectively. Means ± s.d. (n = 3). LY: LY294002; AktI: Akt inhibitor VIII.

TCR/CD28 co-stimulation activates CD28 response element (CD28RE) and nuclear factor-kappa B (NF-κB) that can recruit the histone acetyltransferase PCAF (p300/CREB-associated factor) to the *IL*-2 promoter^33^. Moreover, Nef by itself has been reported to activate NF-κB in T cells^21^. Here, we observed that rNef activated CD28RE and NF-κB (Fig. 3D). This activation was increased by TCR triggering and blocked by the Akt inhibitor VIII (Fig. 3D), suggesting that Nef participates to NF-ĸB and CD28RE activation via Akt phosphorylation. Recombinant Nef together with TCR stimulation, but not rNef alone, stimulated IL-2 production (p = 0.05, anti-CD3 alone vs. anti-CD3 + rNef) (Fig. 4A,B) and T-cell proliferation (p = 0.006, anti CD3 alone vs. anti-CD3 + rNef) (Fig. 4C) that were significantly inhibited by the Akt inhibitor VIII (IL2 production: p = 0.05 at 25 μM; p = 0.01 at 50 μM and T cell proliferation: p = 0.001 at 25 μM; p = 0.002 at 50 μM) and the PI3K inhibitor LY294002 (IL2 production: p = 0.008 at 25 μM; p = 0.008 at 50 μM and T cell proliferation: p = 0.003 at 25 μM; p = 0.001 at 50 μM) (Fig. 4D,E). The enhancement of proliferation and IL-2 production in PBLs was Nef-specific, since it was totally blocked by a neutralizing anti-Nef antibody (Fig. 4B,C). Altogether, our results show that *in vitro* exogenous Nef activates Akt especially in TCR-stimulated PBLs resulting in increased IL-2 production and T-cell proliferation (Fig. 4). In addition, we found a significant difference in the levels of IL-2 and T cell proliferation in CD3 alone stimulated cells and cells stimulated with CD3 in addition to rNef treatment (Fig. 4D,E).

### Protease inhibitors (PI) block Akt activation in PBLs treated with rNef *in vitro*

**Figure 5:**
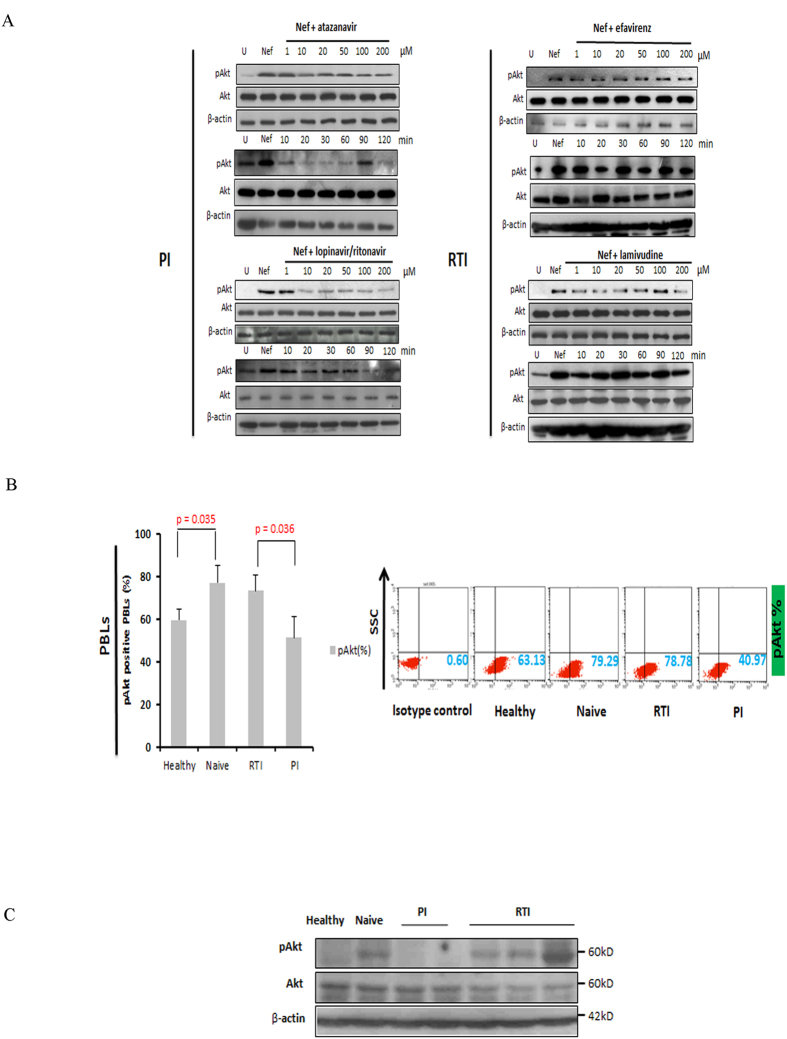
HIV-1 protease inhibitors block Akt activation in rNef-treated PBLs *in vitro* and *ex vivo* in PBLs isolated from cART-treated patients. (**A**), HIV protease inhibitors, but not RTIs, inhibit pAkt(Ser473) activation in a dose dependent manner in PBLs treated with rNef as determined by western blot. Five million PBLs were treated with rNef (100 ng/ml) for 30 min. Expression of pAkt(Ser473), total Akt and β-actin was determined by western blotting (n = 8). (**B**), Decreased Akt activation in PBLs from PI-treated patients compared to RTI-treated and cART naive patients as measured by flow cytometric analysis. Mean values ± s.d. (n = 5). (**C**), Decreased Akt activation in PBLs from PI-treated patients compared to RTI-treated and cART naive patients as measured by western-blotting.

Since HIV-1 protease inhibitors (PI), but not reverse transcriptase inhibitors (RTI), have been reported to block Akt activation in several cell types such as peripheral blood mononuclear cells (PBMCs) and monocytes/macrophages^34^,^35^,^36^,^37^, we assessed the potential role of PIs as immunomodulators in addition to their antiviral effect in HIV-1 infection. *In vitro*, we observed that PIs such as atazanavir and lopinavir/ritonavir inhibited Akt hyperactivation in a time dependent manner (Fig. 5A). Akt activation does not seem dose-dependent upon treatment with lopinavir/ritonavir.

In contrast, RTI such as efavirenz (NNRTI) and lamivudine (NRTI) did not significantly modify rNef-mediated Akt hyperactivation (Fig. 5A). After treatment with lamivudine we did not find any statistically significant decrease in Akt activation as measured by phosphoimager (p > 0.05) (data not shown). At the concentrations of anti-HIV drugs used, more than 80 to 90% of treated cells were viable after two hours of treatment (data not shown).

### Decreased Akt activation and limited IL-2 production and T-cell proliferation are observed in PBLs isolated from HIV-1 patients treated with PI

**Figure 6:**
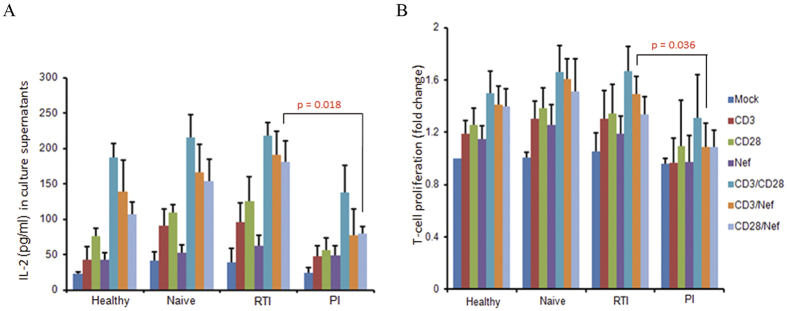
Reduced IL-2 production and T-cell proliferation in PBLs isolated from PI-treated patients. (**A**,**B**), Lower IL-2 production (**A**) and decreased proliferation of PBLs (**B**) in PI-treated patients compared to RTI-treated and cART naive patients, respectively. PBLs were isolated from healthy, naïve and HIV-1 infected individuals under PI and RTI treatment. Five million PBLs were either left untreated or treated with rNef (100 ng/ml), anti-CD3-mAb (5 μg/ml), anti-CD28-mAb (5 μg/ml), anti-CD3-mAb + rNef, anti-CD8-mAb + rNef, and anti-CD3-mAb + anti-CD28-mAb for 24 h. IL-2 production (**A**) and cell proliferation (**B**) was measured using ELISA and MTT cell proliferation assay respectively. Means ± s.d. (n = 5).

To further demonstrate the critical role of Akt in HIV-1 pathogenesis and the potential interest of Akt blockade by PI, we compared Akt activation levels in HIV-1 positive patients treated with PI, RTI or cART naive. Akt activation was higher in cART-naïve patients versus healthy controls (p = 0.035) (Fig. 5B,C). In addition, Akt activation was significantly lower in PBLs isolated from HIV-1 patients treated with PI in comparison to PBLs isolated from HIV-1 patients treated with RTI (p = 0.036) (Fig. 5B,C). In agreement with *in vitro* data (Fig. 4), IL-2 production and T-cell proliferation were significantly lower in PBLs isolated from HIV-1 patients treated with PI in comparison to PBLs isolated from HIV-1 patients treated with RTI (p = 0.018 and p = 0.036, respectively) (Fig. 6A,B).

### Protease inhibitors limit HIV-1 reactivation from T lymphoid cells containing stably integrated HIV-1 or HIV-1 LTR constructs and stimulated with several stimuli including Nef via blocking Akt pathway

Our results showing decreased IL-2 production and T-cell proliferation in PI-treated patients compared to RTI-treated patients suggest that controlling the Akt pathway using PI participates to a lower activation of T cells and thereby could have an impact on HIV-1 recovery from latently-infected T cells^38^,^39^,^40^,^41^,^42^,^43^.

**Figure 7:**
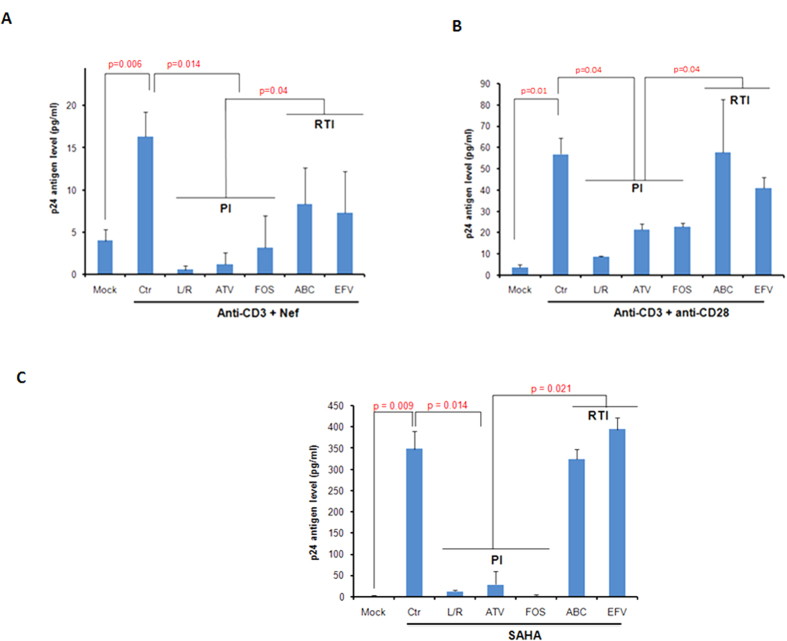
HIV-1 protease inhibitors block HIV-1 recovery from latently infected J-Lat 8.4 cells. (**A**–**C**), J-Lat cells were either left untreated or treated with anti-CD3-mAb (2 μg/ml) +rNef (100 ng/ml) (**A**), anti-CD28-mAb (2.67 μg/ml) +anti-CD3-mAb (2 μg/ml) (**B**) and SAHA (2.5 μM) (**C**) in the absence or presence of different PI and RTI compounds as indicated. The viral production was determined by measuring the level of p24 antigen using ELISA. Means ± s.d. (n = 6). Ctr: Control; L/R, lopinavir/ritonavir; ATV: atazanavir; FOS: fosamprenavir, ABC: abacavir; EFV: efavirenz.

Therefore, we assessed the respective role of PI or RTI treatment on HIV-1 reactivation from latency by measuring viral production in TCR-stimulated latently-infected J-Lat 8.4 cells treated with rNef. J-Lat 8.4 cells have full length HIV-1 minus env and nef genes. We observed that viral reactivation was significantly blocked by treatment with PI, but not with RTI (Fig. 7A). Similarly PI, but not RTI, blocked the activation of viral production by co-stimulation with anti-CD3 + anti-CD28 (Fig. 7B) and by SAHA, a histone deacetylase inhibitor well-known as PI3K/Akt activator^44^,^45^ (Fig. 7C).

**Figure 8:**
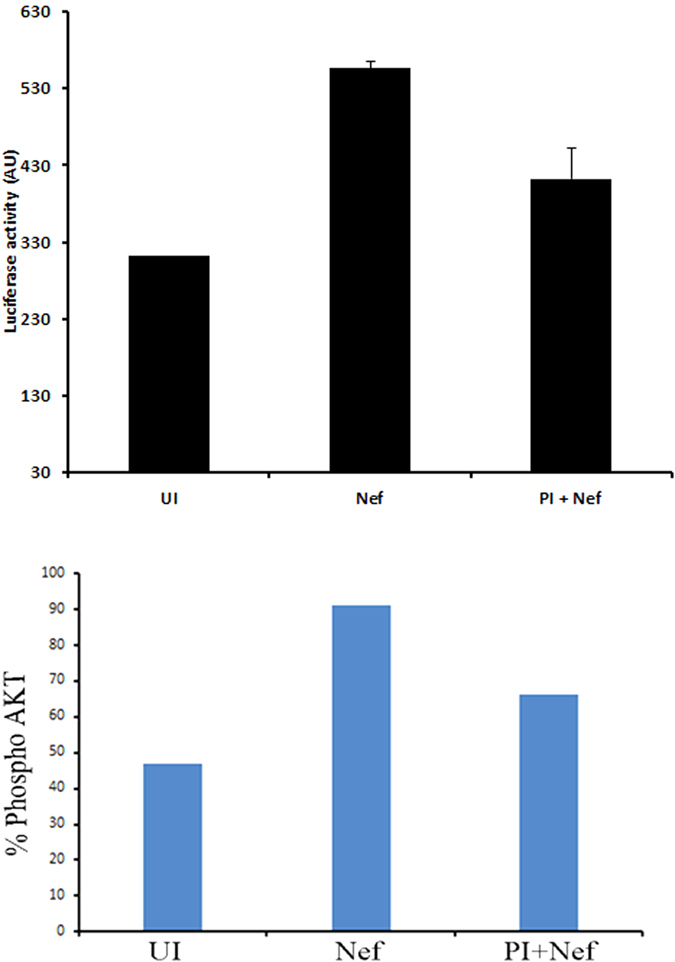
rNef induces HIV-1 expression in a latent model cell line 1G5 and protease inhibitor can subsequently block it. Five million 1G5 cells were either left untreated or treated with rNef in the presence or absence of protease inhibitor. Post 24 h of treatment cells, the expression of luciferase was determined (upper panel) and expression of pAkt was determined by flow cytometry (lower panel). The data shown are representative of two independent experiments.

We then tested the impact of rNef treatment alone on the activation of HIV-1 5′LTR in 1G5 cells, a Jurkat derivate T cell latent model cell line containing stably integrated luciferase gene under the control of the 5′LTR promoter. We found rNef treatment resulted in almost two-fold increase in the expression of luciferase activity as compared to mock treated cells which was subsequently blocked by protease inhibitor (Fig. 8 upper panel). We observed that the Nef-mediated reactivation of HIV-1 from latency in the 1G5 cells was blocked by PI parallel to decreased Akt activation (Fig. 8 lower panel). Our results indicate that PI-mediated blockade of Akt activation could impact the HIV-1 reservoir especially when driven by viral proteins such as Nef.

## Discussion

HIV-1 Nef a pleiotropic early protein has been demonstrated to activate several key signaling molecules including Akt, MAPK and ERK^46^,^47^,^48^. To further add to the previous findings we have demonstrated that HIV-1 Nef acts as a transient adaptor molecule for PI3K and Akt and induces phosphorylation of Akt and subsequent T cell proliferation in *in vitro* and *ex vivo* settings. In addition, we also found that protease inhibitors but not reverse transcriptase inhibitors block Nef-induced Akt phosphorylation resulting in lesser IL-2 production and T-cell proliferation. Most importantly, our results indicate that PI rather than RTI block HIV-1 reactivation from latently infected T-cells especially when stimulated by Nef.

For the first time to our knowledge, we showed that HIV-1 Nef is internalized by CD4+ T cells (Fig. 1C–E) and binds to both Akt and PI3K, suggesting a critical role for Nef as an adaptor protein in the PI3K/Akt pathway (Fig. 2). Our results are consistent with the previously published study where an interaction of Nef and PI3K has been shown^10^. We also demonstrated that Nef colocalizes with Akt in PBLs treated with rNef with the concentration of Nef usually reported in patients (1–10 ng/ml)^49^ (Fig. 1C,D, Supplementary Figs 5 and 6). Nef localization was observed mostly at the cell margins and in the cytoplasm (Fig. 1C,D). On the other hand, expression of Akt was majorly concentrated at plasma membrane and also diffused throughout the cell including nucleus^50^ (Fig. 1C). The mode of internalization and secretion of Nef is still a conundrum. Some studies suggest the involvement of exosomes in intercellular transfer of Nef from infected cells to the bystander cells^51^,^52^,^53^. On the other hand, a recently published study suggests the cell to cell contact rather than exosomes mediated intercellular transfer of Nef^54^. In our study, we found the direct internalization of rNef by the PBLs (Fig. 1E).

Since it has been described that Nef could induce apoptosis in PBLs^49^,^51^ , we quantitated the metabolic activity of rNef treated PBLs at various time points and concentration (1–100 ng/ml) using MTT assay and/or WST-1 assay and monitored cell viability using trypan blue staining (Supplementary Fig. 1A,B). We did not observe any significant decrease in cell viability post rNef treatment. Since results obtained with 100 ng/ml of rNef were more pronounced without significant toxicity (Fig. 1A), we have therefore used this concentration throughout in our experiments. However, we also demonstrated that rNef can induce similar effects at concentration usually reported in the serum of patients (1–10 ng/ml) (Fig. 1A–D and Supplementary Figs 5 and 6)^14^.

The PI3K/Akt pathway is modulated by several pathogenic viruses^15^. We observed that both exogenous as well as endogenous Nef were able to activate Akt in PBLs in both dose dependent (Fig. 1A,C,D,G,H) and time dependent manner (Fig. 1B). Interestingly, we also observed some increase in Akt phosphorylation with increasing dose of HIVΔNef (Fig. 1G). Therefore, we cannot rule out the role of other viral protein(s) contributing to Akt phosphorylation, e.g. the Tat protein (Fig. 1G)^55^.

The role of lipid rafts in intracellular trafficking and signal transduction has been established by several independent studies. Nef interacts with a number of signaling partners including p21-activated kinase-2 in lipid rafts^32^,^56^. Calay *et al.* demonstrated that disruption of lipid rafts negatively influences the activation of Akt at regulatory sites Ser(473) and Thr(308)^57^. In agreement with previous findings, our data show for the first time that Nef and Akt interaction involves lipid rafts (Fig. 3B,C). In addition, we observed also the localization of rNef and pAkt at the cell margins of the PBLs suggesting lipid rafts as a possible site of Nef and Akt interaction (Fig. 1C,D). Several aspects of T cell biology including differentiation and proliferation are modulated by activation of signaling pathways by T cell receptor^29^. In addition, lipid rafts and Akt signaling play a critical role in TCR signaling^58^. We found that in response to stimulation of TCR by anti-CD3-mAb and/or anti-CD28-mAb there was an increase in the activation of Akt. We also observed the synergistic effect of Nef with anti-CD3-mAb and anti-CD28-mAb (Fig. 3A). It seems that Nef can reduce the threshold of CD3/CD28 required for T cell stimulation as shown previously^59^. *In vivo,* exogenous Nef secreted by infected cells can activate uninfected CD4+ T cells present in the vicinity and make them susceptible for infection^60^. In this way, Nef could expand the viral reservoir in infected individual.

Production of IL-2, a cytokine considered as a hallmark of CD4+ T-cell activation, is mediated by the stimulation of NF-kB, NFAT and AP-1 61. Nef has been shown to induce IL-2 production in CD4+ T cells in NFAT and NF-kB dependent manners. Further extending the previous study, we found that rNef activates CD28 responsive element and NF-kB via the Akt pathway which in turn could influence IL-2 production^48^ (Figs 3D and 4A). In addition, Nef exhibited synergism with TCR/CD28 co-stimulation in terms of cell proliferation and IL-2 production mediated by PI3K/Akt pathway (Fig. 4A–E). Our data are in agreement with previous findings^62^,^63^,^64^,^65^. In addition to the previous reports, we found these effects were mediated by Akt/PI3K signaling pathway and were Nef-specific. We observed that, although Nef/anti-CD3 and Nef/anti-CD28 costimulation resulted in higher levels of T-cell proliferation and IL-2 production than treatment with anti-CD3 or anti-CD28 alone, however it was less potent than treatment with anti-CD3/anti-CD28 costimulation (Fig. 4A,B).

Several studies suggest the central role of PI3K/Akt pathway in modulating HIV-1 latent reservoir. For instance, disulfiram is known to deplete phosphatase and tensin homolog (PTEN) protein levels in U1 cells and in resting CD4+ T cells from HIV-negative donors resulting in hyperphosphorylation of Akt and subsequently activation of HIV-1 expression^66^. In another instance, 57704, an agonist of PI3K, reactivates HIV-1 in several latent cell lines^66^. In addition, the role of PI but not RTI in inhibiting Akt activation has been shown in several cell types^34^,^35^,^36^,^37^.

We demonstrated that Nef induced Akt hyperactivation and T-cell proliferation could be effectively inhibited by protease inhibitors but not by reverse transcriptase inhibitors *in vitro* and *ex vivo* (Figs 5 and 6). We found that PI could block Akt activation in both time and dose dependent manners (Fig. 5A,B). The PI nelfinavir has been shown to inhibit Akt phosphorylation by inhibiting proteasomal activity and inducing the unfolded protein response in SQ20B cells^67^.

The choice of treatment (PI versus RTI) has been reported to influence several aspects of HIV-1 pathogenesis^68^. Besides inhibiting polyprotein processing, studies suggest that PI can influence apoptosis^68^ and can block cell to cell dissemination of virions between T cells^69^.

Latency represents the major obstacle in the complete cure of HIV-1 70,71. Several agents targeting various signaling cascades are known to reactivate latent HIV-1 and are termed as latency-reversing-agents (LRAs)^72^,^73^ including activator of PI3K/Akt pathway. In addition, several strategies have been employed in identifying cellular factors involved in viral replication^74^. Indeed Akt inhibitors are suggested as a potential therapeutic molecule against HIV-1 75. We found that protease inhibitors could effectively inhibit the viral reactivation from chronically infected J-Lat cells stimulated with various stimuli (Fig. 7A–C). As indirect evidence we stimulated J-Lat cells with the HDAC inhibitor SAHA which is a potent activator of Akt pathway. We found that PI but not RTI can block the SAHA dependent activation of HIV-1 in latently infected J-Lat cells (Fig. 7C).

To have more direct evidence of the effect of PI on Nef-mediated Akt activation and subsequently on HIV-1 reactivation from latency, we treated Jurkat 1G5 cells, containing an integrated HIV LTR luciferase construct, with protease inhibitor in the presence or absence of rNef. We found that rNef treatment resulted in almost two fold induction in luciferase activity which was subsequently blocked by protease inhibitor (Fig. 8). A two-fold increase in HIV-1 reactivation from 1G5 cells treated with rNef, is in agreement with previously described two-fold increase in NF-kB-mediated LTR activation in U937 cells transfected with LTR-Luc and also two-fold increase of p24 levels in supernatants of chronically HIV-infected U1 cells treated with rNef^60^. Since decreased HIV reactivation in Nef-stimulated 1G5 cells treated with PI parallels decreased Akt activation (Fig. 8, lower panel), our results indicate that PIs have a negative impact on HIV reactivation from latency triggered by Nef protein and Akt activation. Altogether our findings suggest the role of Nef in regulation of HIV-1 latency at physiological level which cannot be compared with strong latency reversal agents such as HDAC inhibitors. In addition, we have determined the levels of pAkt in the blood of naïve, PI and RTI treated patients. We found low levels of pAkt in the patients treated with PI as compared to RTI (Supplementary Fig. 5) further support our findings.

## Conclusion

Our study shows that exposure of T cells to the viral protein Nef leads to Akt activation, which is a critical phenomenon that favors IL-2 production and cellular proliferation. Importantly, we observed that the immunomodulatory effects of PI, by blocking Akt activation especially triggered by Nef, limit HIV-1 recovery from latently-infected T cells. Altogether, our results support the need to further assess the therapeutic use of HIV-1 protease inhibitors and its impact on persistence of HIV-1 reservoirs.

## Materials and Methods

### Study Approval

Ethical approval was granted by the human subject ethics committees of the Saint-Pierre University Hospital and the Besançon University Hospital (CPP EST-2), and written informed consent was obtained from the patients. In addition, all experiments were carried out in accordance with the approved guidelines and regulations (Declaration of Helsinki).

### Isolation and culture of PBMCs and PBLs

Human PBMCs and purified PBLs were prepared from the peripheral blood of healthy donors and cultured as described previously^76^.

### Recombinant Nef treatment

PBLs (5 × 10^6^ cells) were treated with recombinant myristoylated Nef protein (rNef) from the SF2 HIV-1 strain (100 ng/ml) (cat # PR-382, Jena Bioscience, Jena, Germany). Cell pellets were collected at various times after rNef treatment, washed extensively and either lysed before western blot analysis or fixed with BD Cytofix (BD Biosciences, San Jose, CA) for 20 min before flow cytometric analysis. Some PBLs were treated with the PI3K inhibitors LY294002 (0, 25 μM, 50 μM) or Wortmannin (0, 0.5 μM, 1 μM) (Cell Signaling Technology, Beverly, MA), or with the Akt inhibitor VIII (cat # sc-202048A) (0, 25, 50 μM) (Santa Cruz Biotechnology, Santa Cruz, CA) for two hours before addition of rNef (100 ng/ml) for 30 min.

### Confocal microscopy

Five million PBLs cells were incubated with the varying concentration of rNef (1–10 ng/ml) for a period of 30 min. Purity of PBLs cells were assessed by flow cytometry. Cells were washed three times with 1X PBS containing 2% fetal bovine serum followed by fixation in 4% PFA prepared in 1X PBS for 15 min at room temperature. Cells were then washed and permeabilized in 0.1% triton-100 in PBS for 10 min at room temperature followed incubation in primary antibodies: mouse anti-HIV-1 Nef (cat# 65905, Santa Cruz Biotechnology, TX, USA), rabbit anti-Akt1 (cat# 1618, Santa Cruz Biotechnology, TX, USA), rabbit anti-pAKT(Ser473) (cat# 4060, Cell Signaling Technologies, MA, USA) for 1 h at room temperature. Cells were washed and incubated in secondary antibodies: anti-Mouse FITC (cat# 50-010, Argene, FR), anti-Rabbit TRITC (cat# 6718, Abcam, UK) for 1 h at room temperature. Cell pellet was finally dissolved in 10 μl of 1X PBS containing DAPI (Sigma-Aldrich, USA) and placed on slide and mounted with fluorescent mounting media (cat # S3023, DAKO). Cells were observed under Olympus confocal microscope using 405 nm, 488 nm and 543 nm excitation lasers.

### Immunoprecipitation assay

PBLs were either left untreated or were treated with HIV-1 rNef (100 ng/ml) for 30 min. The cell lysates were pre-cleared by adding 50 μl of protein A magnetic beads (Millipore SAS, Molsheim, France) for 1 h at 4 °C. The cleared supernatants were removed, combined with 10 μg/ml anti HIV-1 Nef-Ab or isotype control antibody (Chemicon/Millipore SAS, Molsheim, France) and incubated overnight at 4 °C. The lysates were further incubated with 50 μl protein A magnetic beads (Millipore) at 4 °C for 2 h. Immune complex were washed in the presence of protease inhibitors (Roche, Meylan, France) and bound proteins were eluted with sample buffer and run on 10% SDS-PAGE gels.

### Pull-down assay

HIV-1 Nef (1–210), N-terminus (1–60) and C-terminus (55–210) were cloned in pGEX4T-1 (kindly provided by Dr. Fackler, University of Heidelberg, Germany). For pull-down assays, each construct was transformed into bacteria (BL21DE from Novagen/Millipore SAS, Molsheim, France). A single colony was inoculated in 5 ml broth containing 50 μg/ml ampicillin at 37 °C, 250 rpm in a shaker overnight. A 300 μl aliquot of overnight bacterial culture was used to inoculate 300 ml LB broth containing 50 μg/ml ampicillin and grown for 2.5 h at 37 °C in a shaker (until OD_600_ = 0.5). IPTG was added at a final concentration of 0.1 mM and the culture was grown for an additional 3 h at 37 °C at the shaker. The bacteria were pelleted, washed with ice cold TBS and lysed in bacterial protein extraction reagent (Thermo Fisher Scientific, Rochester, NY). The expressed proteins were immobilized on glutathione agarose beads (Thermo Fischer Scientific) and washed five times with 1 × PBS. Twenty-five micrograms were incubated overnight at 4 °C with 1500 μg of PBL lysate. The suspension was then washed three times in PBS, denatured and analyzed by SDS-PAGE/autoradiography. Input corresponds to 10% of the material used for pull-down.

### Western blot to detect Nef-induced signaling

Cellular extracts from rNef-treated PBLs or PBLs isolated from the peripheral blood of HIV-1 infected subjects were used to examine Akt, PI3K and Nef proteins expression by western blotting as described previously^60^. Anti-Akt-mAb (cat # 2967S, Cell Signaling Technologies, St Quentin, France), anti-PI3K p85 mAb (Cell Signaling Technology, cat # 4292S), anti-Nef-mAb (cat # sc-65905, Santa Cruz Biotechnology), anti-pAkt (Thr308) mAb (cat # 2965S, Cell Signaling Technology), anti-pAkt (Ser473) mAb (cat # 4060S, Cell Signaling Technology), anti-flotillin (Sigma-Aldrich, St. Louis, MO) and anti-β-actin (Sigma-Aldrich) were used. Antiretroviral drugs (atazanavir, lopinavir/ritonavir, efavirenz, lamivudine) were obtained from the hospital pharmacy of CHRU Besançon. PBLs were treated for different periods of time with these drugs at a final concentration of 50 μM. The level of pAkt/Akt was determined by immunoblotting.

For the detection of Akt, pAkt and Nef proteins in lipid rafts, PBLs were incubated with rNef (100 ng/ml) for various periods of time, then washed extensively and treated with a lysis buffer containing 1% Triton X-100. Cell lysates were ultra-centrifuged over a sucrose gradient (100.000 × g) for 16 hours at 4 °C. Lipid rafts are localized in the light fractions (1–3) as described previously^77^. SDS-PAGE was performed using 20 μl of the pooled fractions (1–3). Proteins were then transferred to nitrocellulose membranes that were blocked and revealed with antibodies directed against Akt, pAkt, Nef or flotillin-1-enriched lipid rafts.

### Electrophoretic mobility shift assay

Following stimulation of PBLs with CD3 mAb (5 μg/ml) (cat# 555336), CD28 mAb (5 μg/ml) (cat# 555725) (BD Pharmigen, San Diego, CA) and/or rNef protein (100 ng/ml) for 30 minutes, electrophoretic mobility shift assays (EMSA) were carried out to measure CD28 responsive element (CD28RE) and NF-κB activation as described previously^60^. The complexes were analyzed on a 6% native polyacrylamide gel.

### Cell culture and transfection assays

293T cells were grown in DMEM supplemented with 10% fetal bovine serum (FBS), 100 IU of penicillin/ml and 100 μg of streptomycin/ml (Invitrogen, Carlsbad, CA). Wild-type and nef-deleted proviral infectious clones of NL4-3 virus isolates (HIV-1_pNL4−3_WT and HIV-1_pNL4−3_∆Nef) were kindly provided by Dr Serge Benichou (Institut Cochin, Paris France). Replication competent viruses were produced in 293T cells by the calcium phosphate transfection method (OZ Biosciences, Marseille, France). 5 × 10^6^ PBLs were infected various doses of p24 of either wild-type or ∆Nef virus infectious clones. Cell lysates were prepared and levels of Akt and pAkt were determined by immunoblotting and flow cytometry.

### IL-2 production and T-cell proliferation

PBLs were either left untreated or treated with Akt inhibitor VIII (25 μM, 50 μM) or PI3K inhibitor LY294002 (25 μM, 50 μM) for two hours followed by treatment with rNef (100 ng/ml), anti-CD3 mAb (5 μg/ml), anti-CD28 mAbs (5 μg/ml) for 24 h and levels of intracellular IL-2 and IL-2 production in culture supernatants were measured using flow cytometric analysis (FACSCalibur flow cytometer, BD Biosciences, San Jose, CA) and ELISA (Bender MedSystems cat # BMS221HS, Paris, France) and cell proliferation was measured using the MTT cell proliferation assay kit (cat # 10009365, Cayman Chemical, Ann Arbor, MI).

### FACS analysis

PBLs were fixed and permeabilized (BD Cytofix /Cytoperm kit) (BD Biosciences). Briefly, 2.5 × 10^6^ PBLs were thoroughly resuspended in 250 μl of BD Cytofix/Cytoperm solution for 20 min at 4 °C. Cells were washed two times in 1X BD Perm/Wash solution. The cells were pelleted by centrifugation. The PBLs were thoroughly resuspended in 500 μl of 1X BD Per/Wash containing BD PhosflowPE-conjugated anti-pAkt (cat# 558275)/anti-Akt (cat# 560049) antibodies (BD Biosciences) or isotype control antibody (cat# 551436) (BD Pharmingen). The cells were incubated at 4 °C for 30 min in the dark. The cells were washed three times in 1X BD Perm/Wash solution and resuspended in 1X PBS prior to flow cytometric analysis.

### MTT cell assay

Cell viability was measured using the MTT assay kit (Cayman Chemical). PBLs were seeded at a density of 0.1 × 10^6^/well in triplicates in 96-well plates in a final volume of 100 μl medium containing antiretroviral drugs (50 μM). After 0-2 hrs, 10 μl MTT reagent was added to each well and the plates were incubated for another 4 h in the cell culture incubator at 37 °C. The plate was centrifuged at 500 g for 10 min. The cell culture media was aspirated, 100 μl crystal dissolving solution was added to each well and the absorbance was measured at 570 nm using Multiskan Ex (Thermo Electron Corporation, Cergy-Pontoise, France).

### Reactivation from latency in J-Lat 8.4 cells

The T-lymphoid cell line J-Lat 8.4 was obtained from the AIDS Research and Reference Reagent Program (National Institute of Allergy and Infectious Disease [NIAID], National Institute of Health [NIH]). J-Lat 8.4 cells were mock-treated or treated with anti-CD3 (2 μg/ml), anti-CD28 (2.67 μg/ml) and/or rNef (100 ng/ml), or with SAHA (2.5 μM) in the absence or presence of the different PI and RTI compounds. At 24 h or 48 h post treatment, culture supernatants were harvested and HIV-1 production was measured by determining p24 antigen concentration by ELISA (Innogenetics)^45^. A WST 1 assay (Roche) was performed to assess the viability of treated cells.

### Luciferase assay

Five million 1G5 cells were either left untreated or treated with protease inhibitor and/or rNef for 24 h. After 24 h cells were collected and luciferase expression was determined using promega luciferase assay kit as per manufactures instructions.

### Statistical analyses

The figures show the means and standard deviations for independent experiments. Statistical comparison was performed using the Mann-Whitney U tests (SPSS Inc.; Chicago; USA) or student t test. The level of significance was set at p < 0.05.

## Additional Information

**How to cite this article**: Kumar, A. *et al.* Tuning of AKT-pathway by Nef and its blockade by protease inhibitors results in limited recovery in latently HIV infected T-cell line. *Sci. Rep.* **6**, 24090; doi: 10.1038/srep24090 (2016).

## Supplementary Material

Supplementary Information
