# Supplementary material for: Tuning of AKT-pathway by Nef and its blockade by protease inhibitors results in limited recovery in latently HIV infected T-cell line
Source: Sci Rep. 2016 Apr 14;6:24090. doi: 10.1038/srep24090 (PMC4831010; doi:10.1038/srep24090)
Supplement: Supplementary Information [file srep24090-s1.pdf]

Tuning of AKT-pathway by Nef and its blockade by protease inhibitors results in limited recovery in latently HIV infected T-cell line

Amit Kumar[1+], Wasim Abbas[1+], Laurence Colin[2+], Kashif Aziz Khan[1], Sophie Bouchat[2], Audrey Varin[1], Anis Larbi[3], Jean-Stéphane Gatot[2], Kabamba Kabeya[4], Caroline Vanhulle[2], Nadège Delacourt[2], Sébastien Pasquereau[1], Laurie Coquard[1], Alexandra Borch[5], Renate König[5,6], Nathan Clumeck[4], Stephane De Wit[4], Olivier Rohr[7], Christine Rouzioux[8], Tamas Fulop Jr[3], Carine Van Lint[2++*], Georges Herbein[1++*]

[1] Department of Virology, Pathogens & Inflammation Laboratory, University of Franche-Comté and COMUE Bourgogne Franche-Comté University, UPRES EA4266, SFR FED 4234, CHRU Besançon, Besançon, France, [2] Laboratory of Molecular Virology, Institut de Biologie et de Médecine Moléculaires (IBMM), Université Libre de Bruxelles (ULB), Gosselies, Belgium, [3]Department of Medicine, University of Sherbrooke, Sherbrooke, Canada, [4]Department of Infectious Diseases, CHU St-Pierre, ULB, Bruxelles, Belgium,[5]Research Group "Host-Pathogen Interactions", Paul-Ehrlich-Institute, Langen, Germany; [6]Immunity and Pathogenesis Program, Sanford Burnham Prebys Medical Discovery Research Institute, La Jolla, CA; German Center for Infection Research (DZIF), Langen, Germany,[7]Institut de Parasitologie et Pathologie Tropicale, University of Strasbourg, Strasbourg, France, and [8]Department of Virology, Paris University, EA7327 Paris Descartes, APHP Necker Hospital, Paris, France.

* Correspondence to: Dr Georges Herbein, Department of Virology, University of Franche-Comté and COMUE Bourgogne Franche-Comté University, CHRU Besancon, Hôpital Saint-Jacques, 2 place Saint-Jacques, F-25030 Besançon cedex, France Ph. +33 381 218 877; Fax + 33 381 665 695; E-mail: georges.herbein@univ-fcomte.fr
Or Dr Carine Van Lint, Laboratory of Molecular Virology, Institut de Biologie et de Médecine Moléculaires (IBMM), Université Libre de Bruxelles (ULB), rue des Profs Jeener et Brachet 12, 6041 Gosselies, Belgium. Ph. +32 2650 98 07; Fax + 32 2650 98 00; E-mail: cvlint@ulb.ac.be
[+] AK, WA and LC, [++] GH and CVL contributed equally to the work.
Running Head: HIV and Akt signaling in T-cells

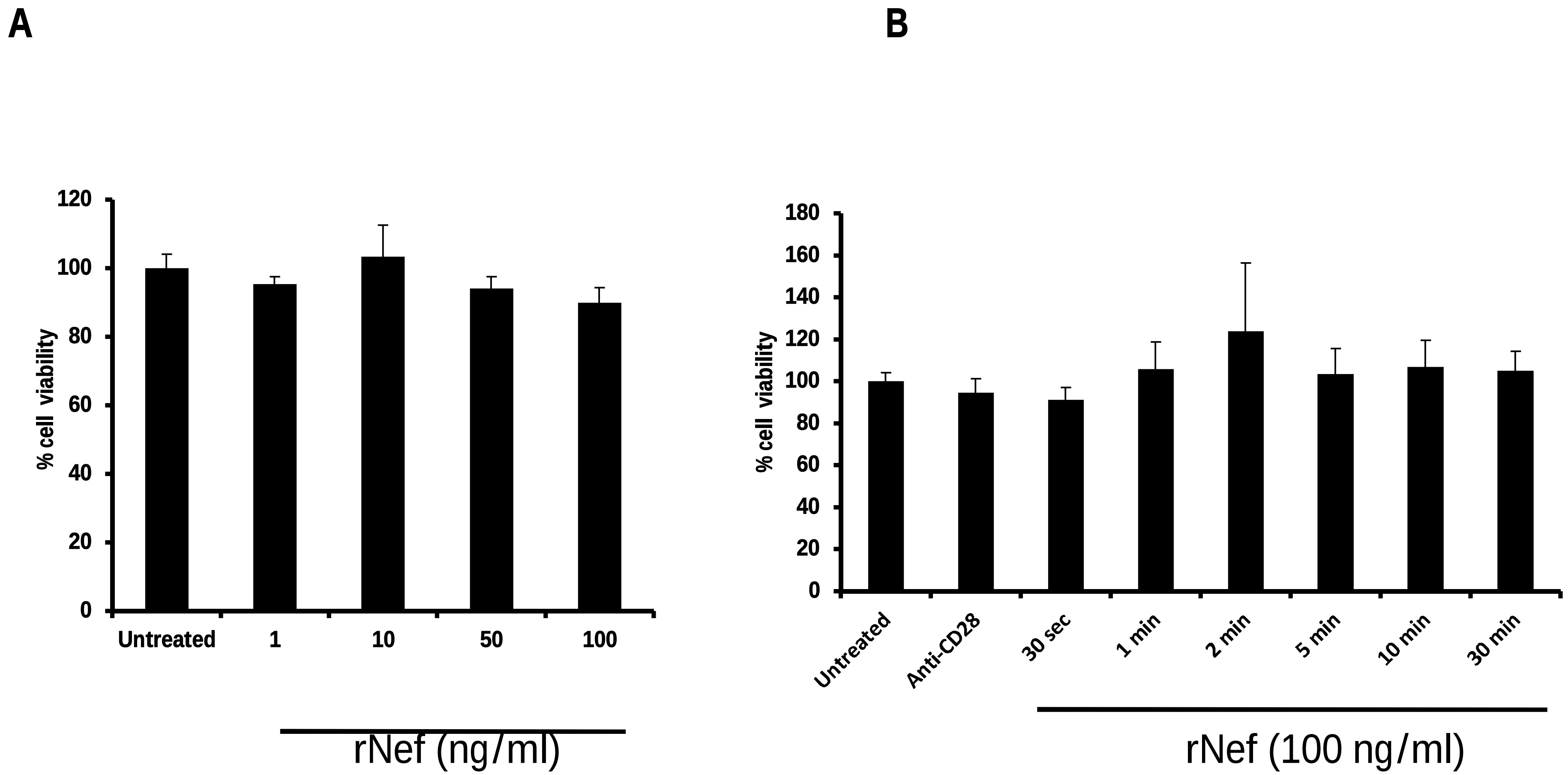

Supplementary Figure 1

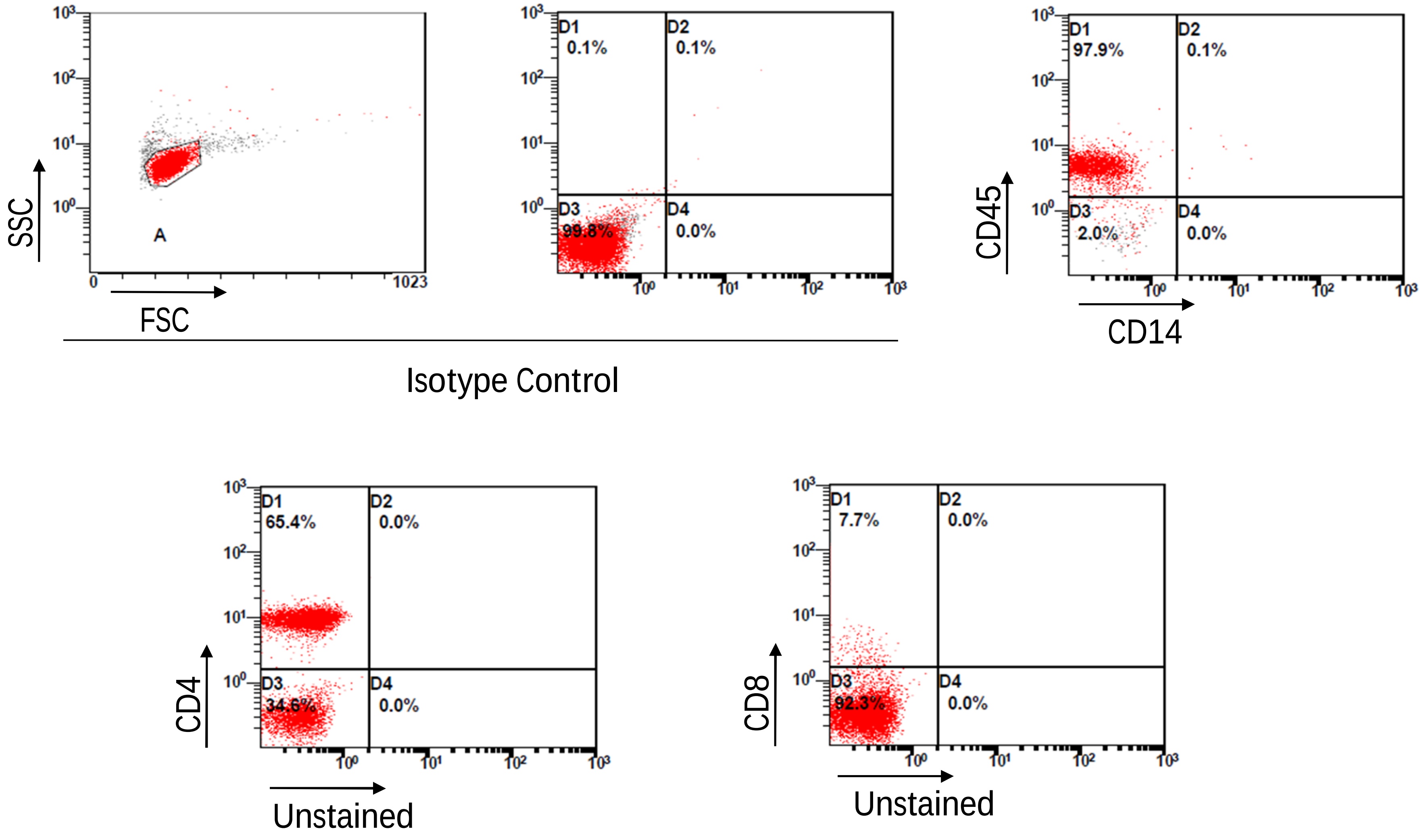

Supplementary Figure 2

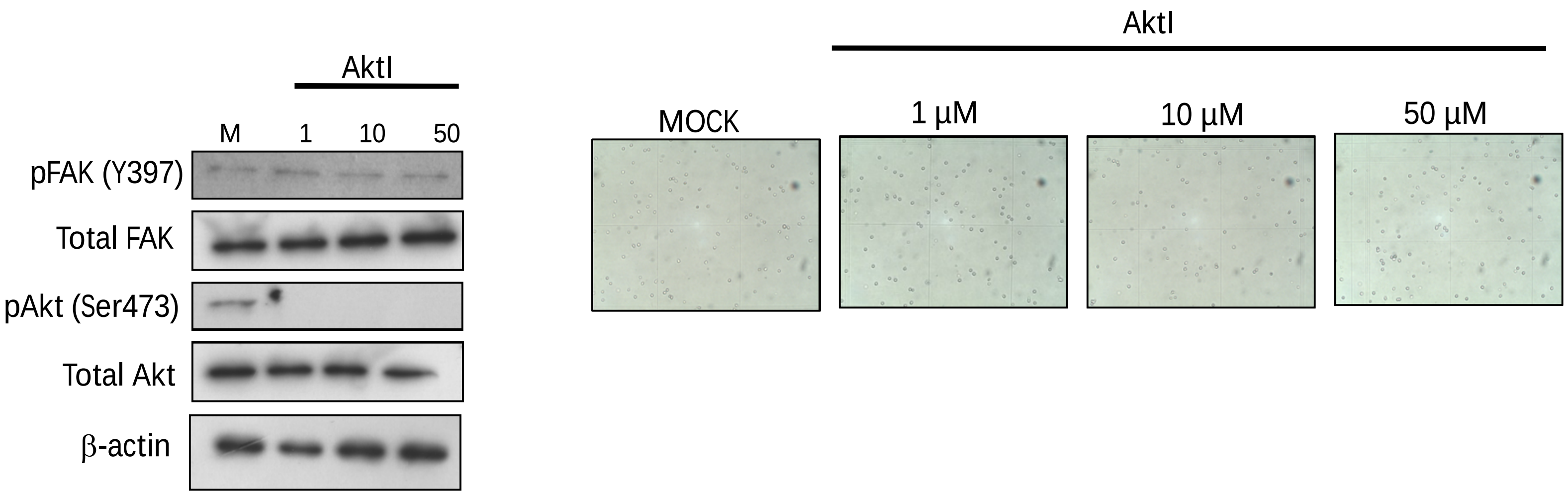

Supplementary Figure 3

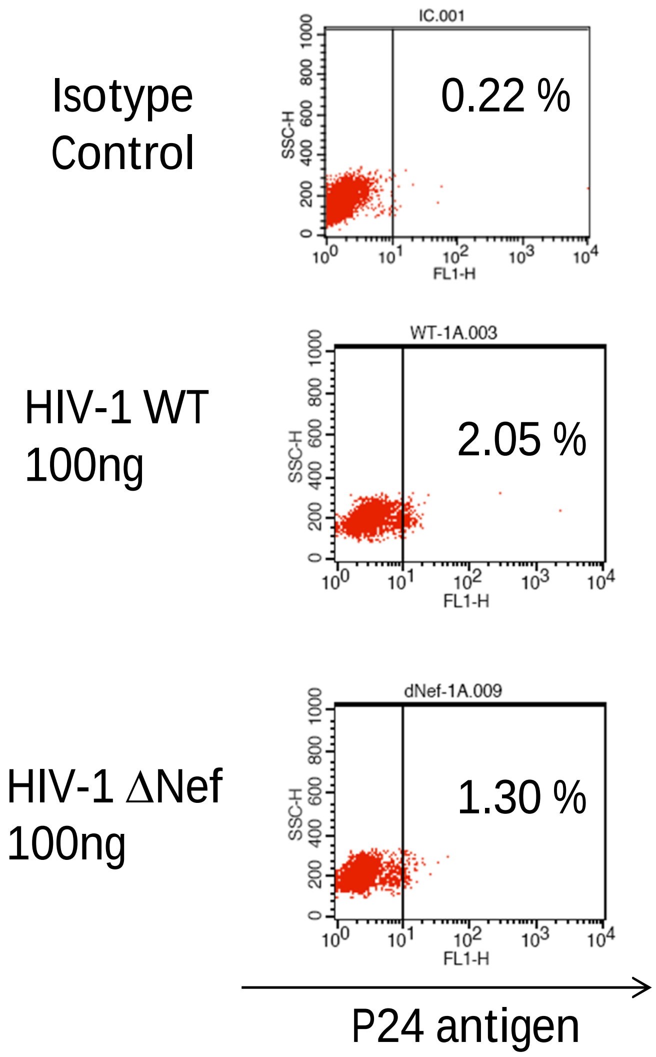

Data obtained from CD4+ T cells 24 hours after infection

Supplementary Figure 4

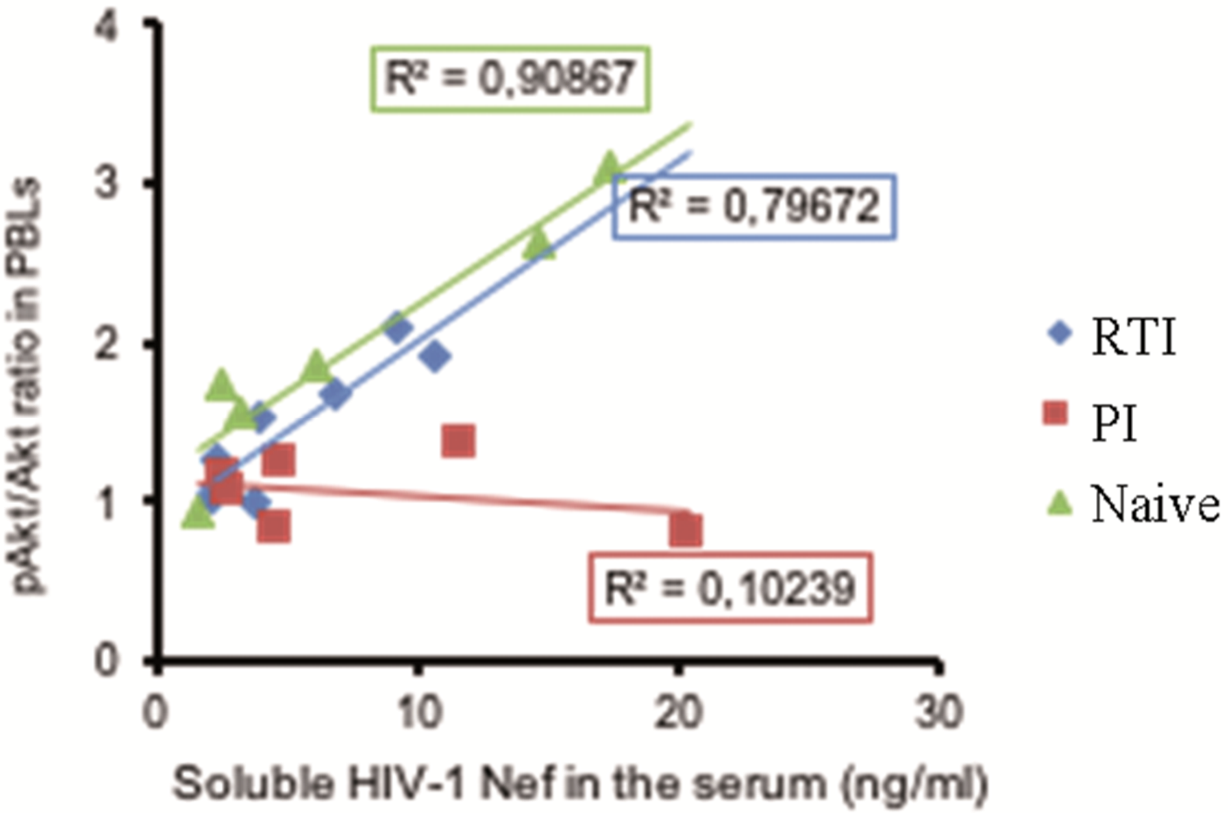
R² = 0,90867
R² = 0,79672
R² = 0,10239
pAkt/Akt ratio in PBLs
Soluble HIV-1 Nef in the serum (ng/ml)
0
1
2
3
4
0
10
20
30
RTI
PI
Naive

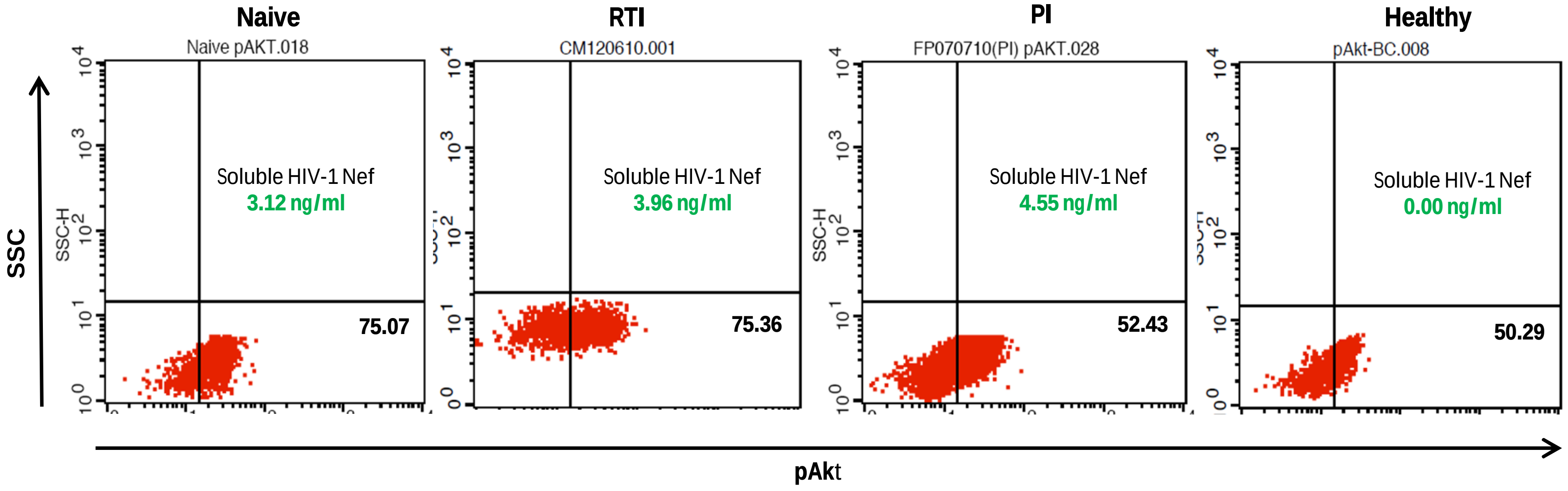

Supplementary Figure 6
